# Peripheral Organ Injury After Stroke

**DOI:** 10.3389/fimmu.2022.901209

**Published:** 2022-06-01

**Authors:** Jin Wang, Jiehua Zhang, Yingze Ye, Qingxue Xu, Yina Li, Shi Feng, Xiaoxing Xiong, Zhihong Jian, Lijuan Gu

**Affiliations:** ^1^ Central Laboratory, Renmin Hospital of Wuhan University, Wuhan, China; ^2^ Department of Anesthesia, Renmin Hospital of Wuhan University, Wuhan, China; ^3^ Department of Stomatology, Renmin Hospital of Wuhan University, Wuhan, China; ^4^ Department of Neurosurgery, Renmin Hospital of Wuhan University, Wuhan, China

**Keywords:** stroke, peripheral organ injury, lung, heart, kidney, spleen, gastrointestinal tract

## Abstract

Stroke is a disease with high incidence, mortality and disability rates. It is also the main cause of adult disability in developed countries. Stroke is often caused by small emboli on the inner wall of the blood vessels supplying the brain, which can lead to arterial embolism, and can also be caused by cerebrovascular or thrombotic bleeding. With the exception of recombinant tissue plasminogen activator (rt-PA), which is a thrombolytic drug used to recanalize the occluded artery, most treatments have been demonstrated to be ineffective. Stroke can also induce peripheral organ damage. Most stroke patients have different degrees of injury to one or more organs, including the lung, heart, kidney, spleen, gastrointestinal tract and so on. In the acute phase of stroke, severe inflammation occurs in the brain, but there is strong immunosuppression in the peripheral organs, which greatly increases the risk of peripheral organ infection and aggravates organ damage. Nonneurological complications of stroke can affect treatment and prognosis, may cause serious short-term and long-term consequences and are associated with prolonged hospitalization and increased mortality. Many of these complications are preventable, and their adverse effects can be effectively mitigated by early detection and appropriate treatment with various medical measures. This article reviews the pathophysiological mechanism, clinical manifestations and treatment of peripheral organ injury after stroke.

## 1 Introduction

Stroke refers to cerebrovascular damage and focal or widespread brain tissue damage due to a variety of causes, including ischemic stroke and hemorrhagic stroke. Stroke involves brain cell and tissue necrosis and has obvious seasonality, especially during the cold season. Although there are an increasing number of in-depth studies on stroke, few methods can be used to treat stroke (1).

Stroke has the second highest death rate worldwide. Although the mortality rate of stroke has decreased significantly in various countries due to the continuous development of medical technology, the incidence of stroke is on the rise (2). According to incomplete statistics, as of, 2016, there were 67.6 million people suffering from ischemic stroke and 15.3 million people suffering from hemorrhagic stroke. From 2006 to, 2016, the prevalence of ischemic stroke increased by 2.7%, while the prevalence of hemorrhagic stroke decreased by 1.7% (3).

**Figure 1:**
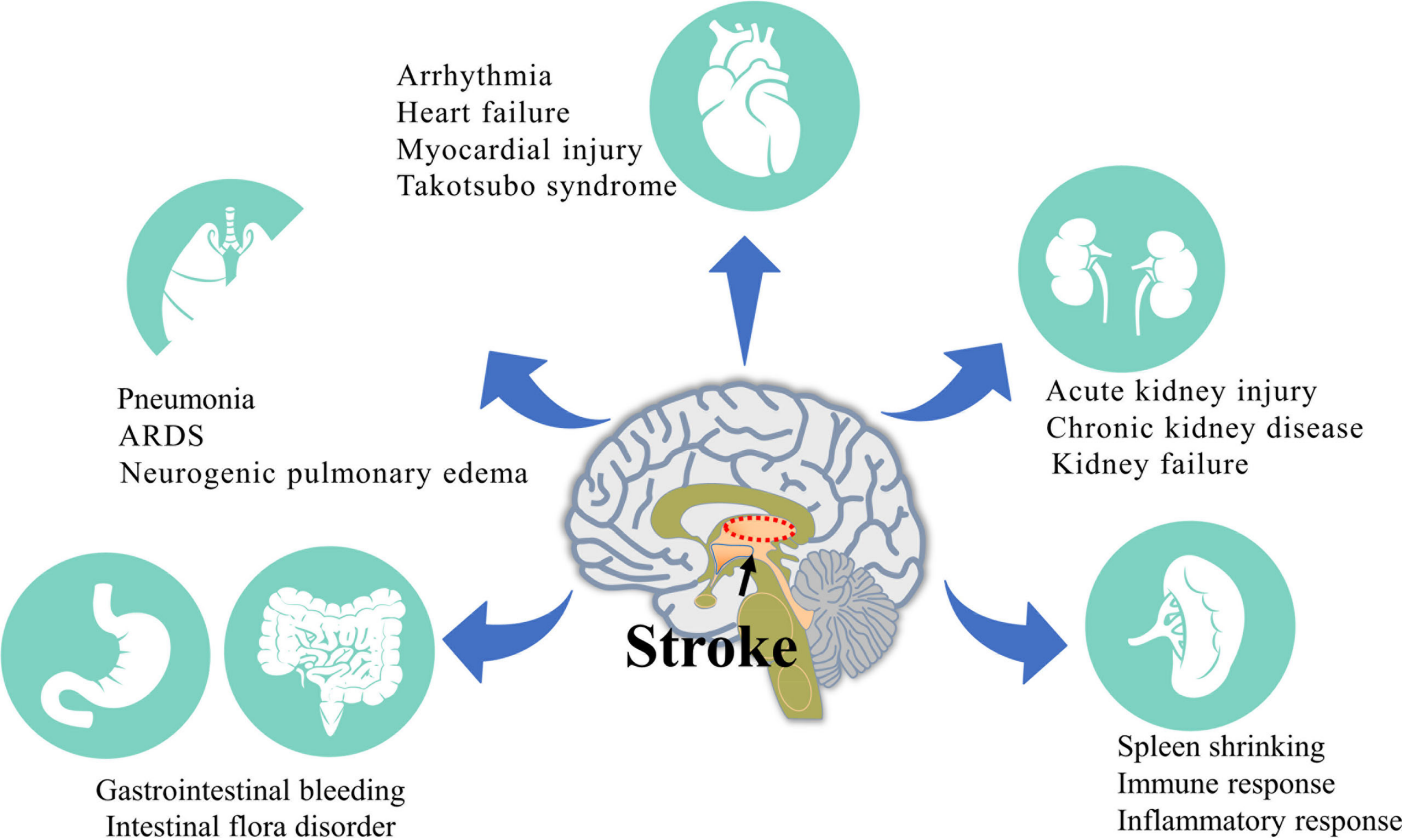
Injury of various peripheral organs after stroke. The activation of sympathetic nerve, hypothalamus-pituitary-adrenal axis and immune system after stroke leads to a series of systemic events and finally leads to the injury of various peripheral organs. The most common peripheral injuries include the Lung, Heart, Gastrointestinal tract, Kidney and Spleen.

The pathophysiological mechanism of stroke is complex, and brain damage usually affects the normal function of peripheral organs and can even cause serious damage (4). At the same time, injury to peripheral organs after stroke often aggravates brain injury and affects patient recovery (**Figure 1**). With a focus on pathophysiology, this review discusses the pathophysiological mechanism underlying peripheral organ injury after stroke and closely links the brain with peripheral organs to identify a more effective and comprehensive method for the treatment of stroke.

### 1.1 Lung Injury After Stroke

Stroke breaks out a strong inflammatory cascade in the brain, but the peripheral immune system is inhibited by immune regulation of compensatory release of neurotransmitters, this phenomenon is defined as stroke-induced immunosuppression (SIIS) (5). The direct consequence of SIIS is to make stroke patients more vulnerable to infections, of which stroke-associated pneumonia (SAP) is the most common and often fatal (6). It has been proved in clinical trials that stroke-induced immunosuppressive syndrome is an independent risk factor for stroke-associated pneumonia (7). Previous studies have shown that older and more severe nerve damage and dysphagia are important factors for pneumonia after stroke (8–12). About 1/10 of stroke patients develop pneumonia in the acute phase (13). The occurrence of post-stroke pneumonia is related to 30-day and 1-year mortality, longer hospital stays and dependence on discharge (14). In addition, neurogenic pulmonary edema (NPE) is also one of the common pulmonary complications after stroke. The mechanism of NPE may be the damage of alveolar capillary barrier caused by a large number of nervous system discharges after severe craniocerebral injury and the lung volume overload caused by the increase of systemic vascular resistance. Due to the lack of effective and timely treatment, NPE is usually associated with higher mortality, which suggests that NPE may be one of the causes of poor prognosis in patients with stroke (15). In this review, we focus on the pathogenesis and emerging treatments of SAP and NPE, and further speculate on the role of immune factors in it.

#### 1.1.1 Pathophysiological Mechanism of Lung injury After Stroke

There are two main theories about the pathophysiological mechanism of SAP: Aspiration Theory and stroke-induced immunosuppression (SIIS) (16). A small amount of inhaled substances caused by dysphagia in stroke patients is a key factor in SAP, which may be related to the abnormal transmission of dopamine (17). An experiment in guinea pigs has shown that blocking D1 dopamine receptors in guinea pigs can inhibit swallowing reflexes and reduce substance P in terminal organs (18). Due to the decrease of dopamine production in substantia nigra and striatum of stroke patients, the expression of substance P in glossopharyngeal nerve and cervical parasympathetic ganglion decreased, resulting in dysphagia (17). The risk of SAP is related to substance P deficiency. Treatment with angiotensin converting enzyme (ACE) inhibitors in stroke patients can increase the level of substance P and reduce the incidence of pneumonia in patients with dysphagia (19).

The main causes of SIIS are the transformation from Th1 phenotype to Th2 phenotype of lymphocytes, the decrease of lymphocytes and NK cells in blood and spleen, and the impairment of defense mechanism of monocytes and neutrophils (5). One of the causes of SIIS may be that stroke activates the sympathetic system and the hypothalamus-pituitary-adrenal axis (20). An experimental study shows that injection of 200 Streptococcus pneumoniae colony-forming units into the nasal cavity of stroke mice can cause pneumonia and bacteremia, while 200000 colony-forming units are needed in fake animals to induce similar diseases. but it can be prevented by adrenoceptor blockers (21). The immunosuppressive state caused by sympathetic activation after stroke is characterized by Th-mediated lymphopenia and functional inactivation of monocytes and Th1 (22), which is more obvious in mice with large cerebral infarction (23). A series of clinical and experimental evidence shows that damaged brain tissue produces a variety of pro-inflammatory cytokines, which can activate the hypothalamus-pituitary axis system, resulting in increased adrenocorticoid secretion and T lymphocyte apoptosis (20).

Neurogenic pulmonary edema (NPE) after acute stroke is an acute respiratory distress syndrome (ARDS), which is characterized by acute onset, obvious infiltration of pulmonary interstitial fluid and rapid regression (15). The pathophysiological mechanism of NPE is the joint participation of nervous system, circulatory system and respiratory system. The occurrence of NPE may be due to the activation of a specific trigger area of the central nervous system located in the brainstem, resulting in excessive sympathetic nerve activation leading to peripheral vasoconstriction, increased systemic vascular resistance (SVR) and enhanced venous reflux. After these changes, the pulmonary capillary hydrostatic pressure (PCP) increased, the alveolar wall was damaged, and fluid and red blood cells infiltrated into the pulmonary interstitium and alveoli to form a typical NPE (24).

#### 1.1.2 Treatment

Post-stroke pneumonia is usually directly associated with bacterial infections, including aerobic Gram-negative bacilli and Gram-positive cocci (25),the most common bacterial infections are Pseudomonas aeruginosa and Staphylococcus aureus (26). Selecting targeted antibiotics according to the type of bacteria infected is an important step in the treatment of SAP, and ampicillin + sulbactam group is a good choice (27). According to the pathophysiological mechanism of SAP, head raising, oral care and dental treatment for stroke patients can effectively improve swallowing function and cough reflex sensitivity, which is the key to the treatment and prevention of SAP. For elderly stroke patients, angiotensin converting enzyme inhibitors can well improve cough reflex sensitivity, thus reducing the risk of SAP (**Table 1**).

**Table 1:** Injury types and treatment of different organs after stroke.

Organs	Types of Injury	Treatment	References
Lung	Pneumonia after stroke	Angiotensin-converting enzyme inhibitors, β-adrenergic receptor blocker, Ampicillin and sulbactam	(21, 27, 28)
Lung	Neurogenic pulmonary edema	Vasoactive substances, diuretics, rehydration, supplement oxygen and mechanical ventilation	(29)
Heart	Arrhythmia	Antiarrhythmic drugs, Pernanent pacemaker implantation, Cardioverter-defibrillator implantation, Urgent coronary angiography, Supportive care and correction of electrolyte abnor- Malities, β-blockers	(30–32)
Heart	Atrial fibrillation	Atrial fibrillation, Antiplatelet	(33)
Heart	High blood pressure	Nicardipine, Nitroprusside, Labetalol, ACEI	(32, 34)
Heart	Heart failure	Antithrombotic treatments	(35)
Heart	Myocardial Infarction	Alteplase, Coronary angioplasty and PCI	(36)
Kidney	Acute kidney injury	Dialysis, Apisaban, Rivasaban, Aspirin	(37–39)
Spleen	Spleen shrinking	Intravenous infusion of human umbilical cord blood cells, Multipotential adult progenitor cells treatment	(40, 41)
Gastrointestinal tract	Gastrointestinal bleeding	Antiplatelet drugs, Misoprostol, Proton pump inhibitors	(42)
Gastrointestinal tract	Intestinal flora disorder	Rhubarb anthraquinone glycosides, Probiotics	(43, 44)

The treatment of NPE mainly includes two aspects. on the one hand, it is based on the treatment of primary central nervous system injury, with emphasis on reducing intracranial pressure to prevent sympathetic nerve discharge, which is considered to be the main culprit of lung injury (45). Another important aspect is supportive treatment, including vasoactive substances, diuretics, rehydration, supplement oxygen and mechanical ventilation if necessary (29). Studies have shown that intravenous injection of 25% albumin does not improve the prognosis of patients with ischemic stroke, but increases the risk of pulmonary edema (46, 47). A study on the treatment of 12 cases of aneurysmal SAH with NPE shows that endovascular treatment of severe SAH with NPE is an effective regimen (48). However, due to the lack of sample size, further research is needed to ensure the reliability of the conclusion.

### 1.2 Heart Injury After Stroke

Adiovascular disease is regarded as the predis-posing risk factor for stroke (49),cardiovascular complications are also the second leading cause of death after stroke (50). In the first few days after stroke, cardiac complications are a common problem, including arrhythmia, heart failure, myocardial injury, non-fatal coronary syndrome, Takotsubo syndrome and other neurocardiogenic syndrome. The study of the interaction between brain and heart has been going on for centuries and has made great progress in the last decade (51), more and more clinical and neuroimaging studies as well as animal studies suggest that this series of cardiac complications may have the same underlying mechanism (52). In this review, we summarize these cardiac complications as stroke-heart syndrome. The main pathophysiological mechanisms include hypothalamus-pituitary-adrenal axis(HPA) (53), the gut dysbiosis (54) and immune and inflammatory response (55), linking the effects of these mechanisms may help us to find new treatments. Some studies have shown that stroke-heart syndrome may originate from the structural or functional changes of central autonomic neural network (CAN) after stroke (52), and affect heart rate and cardiac contractility through sympathetic nervous system and parasympathetic nervous system (56). When brain injury occurs, different areas and degrees of injury will lead to different results. For example, stimulating the orbit of the frontal lobe and anterior cingulate gyrus can affect blood pressure and heart rate, ischemia in the insular cortex may lead to changes in blood pressure and arrhythmias (57). In addition, clinical trials have shown that patients with LDL levels below 70 mg per deciliter have a lower risk of cardiovascular events than patients with LDL levels ranging from 90 mg to 110 mg per deciliter after ischemic stroke. This suggests that LDL may be an independent risk factor for cardiovascular events after stroke (58).

#### 1.2.1 Pathophysiological Mechanism of Heart Injury After Stroke

##### 1.2.1.1 Hypothalamus-Pituitary-Adrenal Axis

The mediator of cortisol is the hypothalamus-pituitary-adrenal axis, and the HPA axis plays a key role in the process of balance in the body (59). Corticotropin-releasing hormone is secreted by the hypothalamic paraventricular nucleus, which stimulates the pituitary gland to release corticotropin, which stimulates the adrenal gland to release the steroid hormone cortisol (60). Elevated cortisol levels increase the mortality of stroke patients (53). In the study of the animal model of middle cerebral artery occlusion (MCAO) in rats, it was found that the activation of paraventricular nucleus was due to the activation of N-methyl-D-aspartate (NMDA) receptor by glutamate, which led to arrhythmia (61). Inhibition of oxidative signals in the paraventricular nucleus can be used as a new method for the treatment of heart failure caused by myocardial infarction (62). Elevated catecholamine levels play an important role in stroke-heart syndrome. Animal studies have shown that plasma catecholamine levels increase after middle cerebral artery occlusion, which may lead to myocardial injury (63). Catecholamine acts on cardiac β-receptors, activating cyclic adenosine monophosphate-protein kinase A signal and increasing intracellular Cyclic adenosine monophosphate (CAMP). CAMP binds to protein kinase A and phosphorylates L-type calcium channels, which makes mitochondrial overload trigger oxidative stress and eventually lead to cardiomyocyte death (50). *In vitro* and *in vivo* studies have shown that the intermediates formed during the oxidation of catecholamines are related to cardiotoxicity (64). The consequence of this increase in catecholamine levels is cardiomyocyte necrosis, hypertrophy and fibrosis, and the production of reactive oxygen species, alteration of the calcium-handling proteins, coronary blood flow redistribution (65).

##### 1.2.1.2 Immuno-Inflammatory Response

Immuno-inflammatory response plays a significant role quickly after stroke and is also an important factor in stroke progression (66). In the early stage of stroke, both innate immunity and acquired immunity are involved in local and systemic inflammation (50). The complex interaction involves a variety of mechanisms. In this article, we focus on the immune and inflammatory responses that mediate stroke cardiac syndrome.

**Figure 2:**
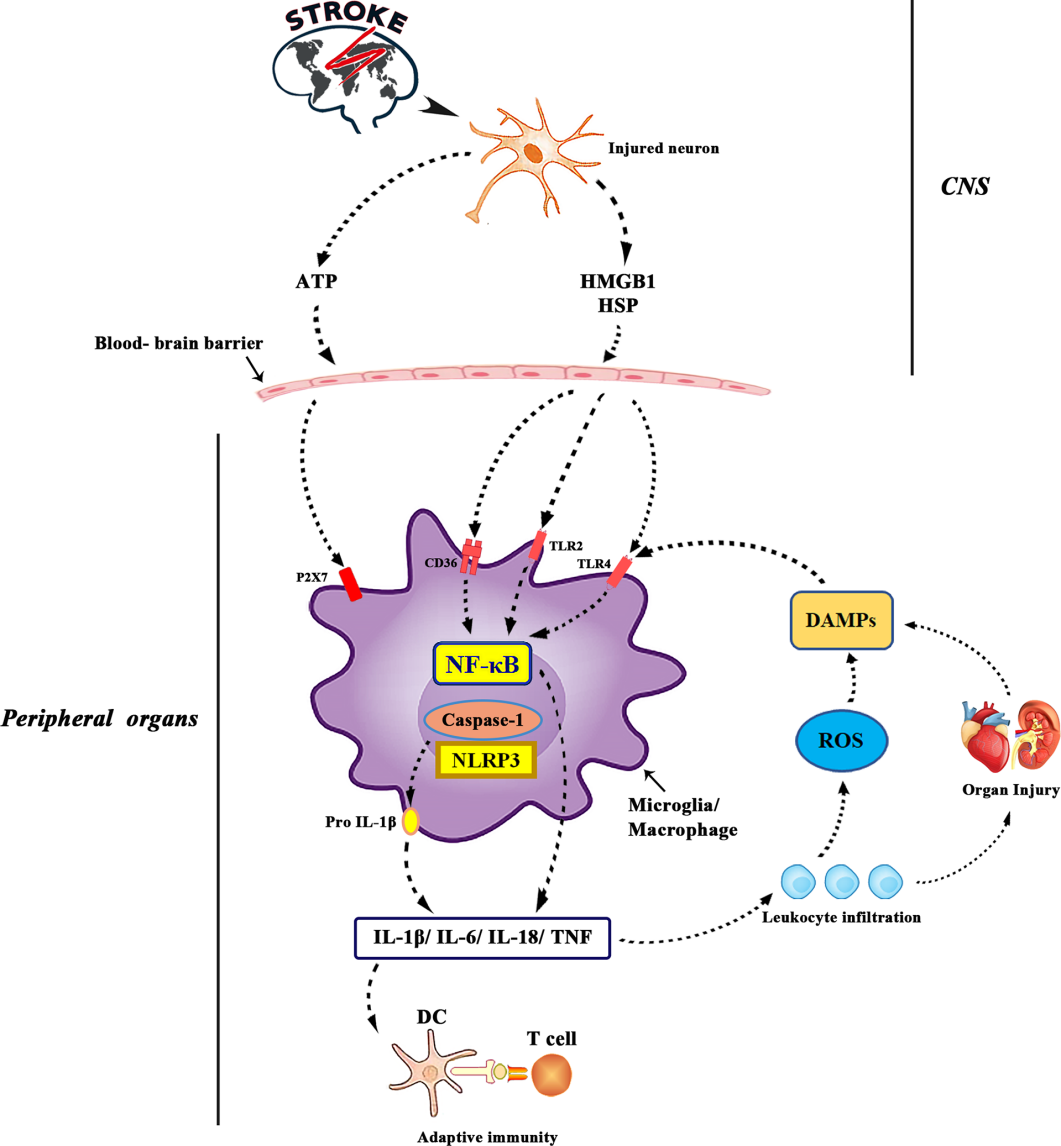
ATP is released from damaged neurons, activating purinergic receptors on microglia and macrophages and leading to the production of pro-inflammatory cytokines. Interleukin-1 converting enzyme (ICE; caspase1) is embedded in a polyprotein complex (NLRP3 or inflammatory body) and activated by P2X7 receptors in microglia. Cell death leads to the formation of DAMPs, which activates TLR, especially TLR2 and TLR4. DAMPs released by ischemia includes high mobility group protein B1, heat shock protein 60 and so on. TLRs binds to scavenger receptors (such as CD36) and up-regulates the expression of inflammatory genes through transcription actor nuclear factor-kB. DAMPs is also produced by matrix decomposition caused by lyases released by dead cells and the effect of reactive oxygen species on lipids. Finally, the production of cytokines and the activation of complement lead to the increase of leukocyte infiltration and tissue damage, which leads to the production of more DAMPs. The antigen revealed by tissue injury was presented to T cells, which laid the foundation for adaptive immunity.

In the early stage of acute brain injury, local inflammatory responses in the brain parenchyma (including microglia proliferation, astrocyte proliferation and cytokine/chemokine secretion) maintain endothelial cell activation. During this period of ischemia, a large number of reactive oxygen species (ROS) are produced in the brain and immune cells. Then, reactive oxygen species can activate endothelial cells, cause oxidative stress and destroy the blood-brain barrier (67). Resident macrophages transform into M1 phenotypes, exacerbating inflammation by releasing pro-inflammatory cytokines (68). After ischemia, the brain releases damage-associated molecular patterns (DAMP), stimulates pathogen recognition receptors Toll-like receptors and TLR-4, and increases the production of pro-inflammatory mediators IL-6, IL-1 β, TNF-α, chemokine and their receptors (**Figure 2**). Through these mediators, the brain recruits peripheral immune cells to the site of brain injury and then crosses the damaged blood-brain barrier (68). Enter the systemic circulation to produce possible secondary heart injury. An animal experimental study reported that abnormal inflammation and apoptotic cells appear in the cerebellum and heart of non-human primate transient global cerebral ischemia (TGI) model (69), suggesting a potential correlation between inflammation and heart injury. Tumor necrosis factor-α leads to ubiquitin and then degradation of troponin I, which eventually leads to a decrease in the contractile function of cardiomyocytes (70). Transforming growth factor-β 1 (TGF-β 1) induces increased expression of IL-6 (71). IL-6 is a cytokine that can regulate the growth, apoptosis and survival of cardiac cells (72). Recent studies have suggested that activation of TLR-4 and elevated levels of IL-1 β are associated with heart failure (73). In patients with ischemic stroke and cerebral hemorrhage, systemic inflammatory response is activated (74). Systemic inflammatory response syndrome is usually characterized by abnormal white blood cell count, increased respiration and heart rate, and abnormal body temperature. This increases the risk of intracranial and systemic complications. The number of CD74+ cells and the expression of CD74mRNA in peripheral blood monocytes increased significantly in patients with ischemic stroke (75). CD74+ cells are mainly distributed on CD4+T cells, monocytes and dendritic cells (75). Monocytes are related to cardiac salvage function damage and poor left ventricular remodeling after acute myocardial infarction (76), and can be used as a new treatment for ischemic brain injury in the future.

##### 1.2.1.3 Intestinal Biological Disorder

More and more experimental studies have shown that there are interactions between intestinal microbiota and central nervous system as well as heart, which are called brain-gut axis and intestine-heart axis. The main function of the gut-blood barrier is to regulate the absorption of nutrients, electrolytes and water, and to prevent pathogenic microorganisms and toxic substances from entering the bloodstream (54). Stroke can lead to the destruction of the intestinal-blood barrier, thereby increasing intestinal permeability, imbalance of intestinal microflora and transfer to the bloodstream (35).Increased intestinal permeability can promote inflammation (54), while systemic inflammation can aggravate cardiac dysfunction, which has been explained above. Trimethylamine N-oxide (TMAO) is an important gut microbe-dependent metabolite synthesized by dietary choline, betaine and L-carnitine (77). TMAO mediates platelet overactivation and thrombosis, and it also enhances inflammation by acting on dendritic cells, macrophages and platelets (77). Changes in TMAO levels are associated with atherosclerosis, myocardial infarction, thrombosis and heart failure (78, 79).

#### 1.2.2 Treatment

The incidence of stroke-cardiac syndrome after stroke is very high, and the damage of cardiac function affects the prognosis and mortality of patients to a great extent. Almost 80% of AIS patients develop hypertension for various reasons. The side effects of severe hypertension increase the risk of cardiopulmonary complications, cerebral hemorrhage and cytotoxic edema, and are associated with adverse outcomes after AIS (80). Drugs such as nicardipine, nitroprusside and labetalol are recommended for the treatment of hypertension in the acute phase of stroke (systolic blood pressure is greater than 180mmHg) (34). β-blockers can not only inhibit sympathetic activation and inflammation, but also prevent chronic remodeling and treat arrhythmias (81), and may reduce heart injury after stroke. Among them, propranolol has been proved to play a neuroprotective effect by blocking the upregulation of IL-6, thus improving the prognosis after brain injury (82). As recommended in the current clinical guidelines (83), secondary stroke prevention with oral anticoagulants can effectively control the risk factors of cardiovascular disease, but there is no good plan for the treatment and prevention of cardiac complications after stroke. With the in-depth study of the pathophysiology of brain-heart interaction, new and more comprehensive treatments may emerge in the future.

### 1.3 Renal Injury After Stroke

Acute renal injury (AKI) is one of the common complications after stroke, which can lead to renal failure (84). Studies have shown that acute renal injury after stroke is associated with higher mortality and poor functional prognosis (85). In a systematic retrospective study of 12,325,652 patients with ischemic stroke, the incidence of acute renal injury (AKI) was 9.6% (86). High score of National Institutes of Health Stroke Scale (NIHSS)and hypertension are important indicators for the incidence of AKI in stroke patients on admission (85). In addition, diabetes, elevated plasma osmotic pressure and the use of cyclic diuretics are also risk factors for AKI after stroke (87–89). Chronic kidney disease (CKD) refers to the damage of kidney structure or function caused by various causes for more than 3 months, which is mainly characterized by a decrease in glomerular filtration rate (GFR), which usually develops with the passage of time and increases the risk of stroke (90). CKD is usually diagnosed and staged by measuring glomerular filtration rate (GFR), creatinine clearance and albuminuria, while lower GFR and albuminuria are associated with poor prognosis after stroke (91, 92), which has been demonstrated in other clinical trials (93) (**Table 2**).

**Table 2:** Clinical trials targeting the peripheral organs injury after stroke.

Organs	Clinical trials	References
Lung	Stroke-associated pneumonia– The Predict study	(7)
Lung	Biomarkers for predicting pneumonia after stroke	(94)
Lung	Comparison of diagnostic utility of SAP	(95)
Lung	ALIAS (Albumin in Acute Ischemic Stroke) Trial	(46, 47)
Heart	COSSACS trial	(96, 97)
Heart	A comparison of two LDL cholesterol targets after Ischemic Stroke	(58)
Heart	AREST trial	(98)
Heart	Direct oral anticoagulants after stroke onset	(99)
Heart	The Insulin Resistance Intervention after Stroke (IRIS) trial	(100)
Heart	Urate predicts subsequent cardiac death in stroke survivors	(101)
Spleen	Post-stroke infections associated with spleen volume reduction	(102)
Spleen	Acute splenic responses in patients with ischemic stroke	(74)
Kidney	Acute kidney injury in acute ischemic stroke patients	(86)
Kidney	Effect of high-dose Atorvastatin on renal function in patients with stroke	(103)
Kidney	The eGFR predicted long-term mortality after ischemic stroke	(93)
Kidney	The URICO-ICTUS trial	(104)
Kidney	The effect of Clopidogrel added to Aspirin on kidney function	(105)
Gastrointestinal tract	Risk score to predict gastrointestinal bleeding after acute ischemic stroke	(42)
Gastrointestinal tract	The NAVIGATE-ESUS trial	(106)
Gastrointestinal tract	Pharyngeal electrical stimulation in the treatment of dysphagia after stroke	(107–109)

There is a strong two-way relationship between stroke and kidney disease, which may be related to their similarities in anatomy, hemodynamics and vascular regulation (110). The glomerular afferent arterioles located in the paramedullary renal artery and cerebral perforating artery in the kidney and brain, respectively, originate directly from the short arterioles and large arteries, and are responsible for maintaining perfusion pressure and blood flow (111). Even if arterial blood pressure is constantly changing, the brain and kidneys can be automatically regulated to maintain a certain range of blood perfusion (84).The pathophysiological interaction between brain and kidney is complex, but many studies have focused on brain dysfunction caused by renal injury. In this review, we focus on the pathophysiological mechanisms of renal injury after stroke, including neuroendocrine system, inflammatory and immune response, extracellular vesicles (EVS) and microRNA(miRNA), as well as related prevention and treatment.

#### 1.3.1 Pathophysiological Mechanism of Renal Injury After Stroke

##### 1.3.1.1 Neuroendocrine System

**Figure 3:**
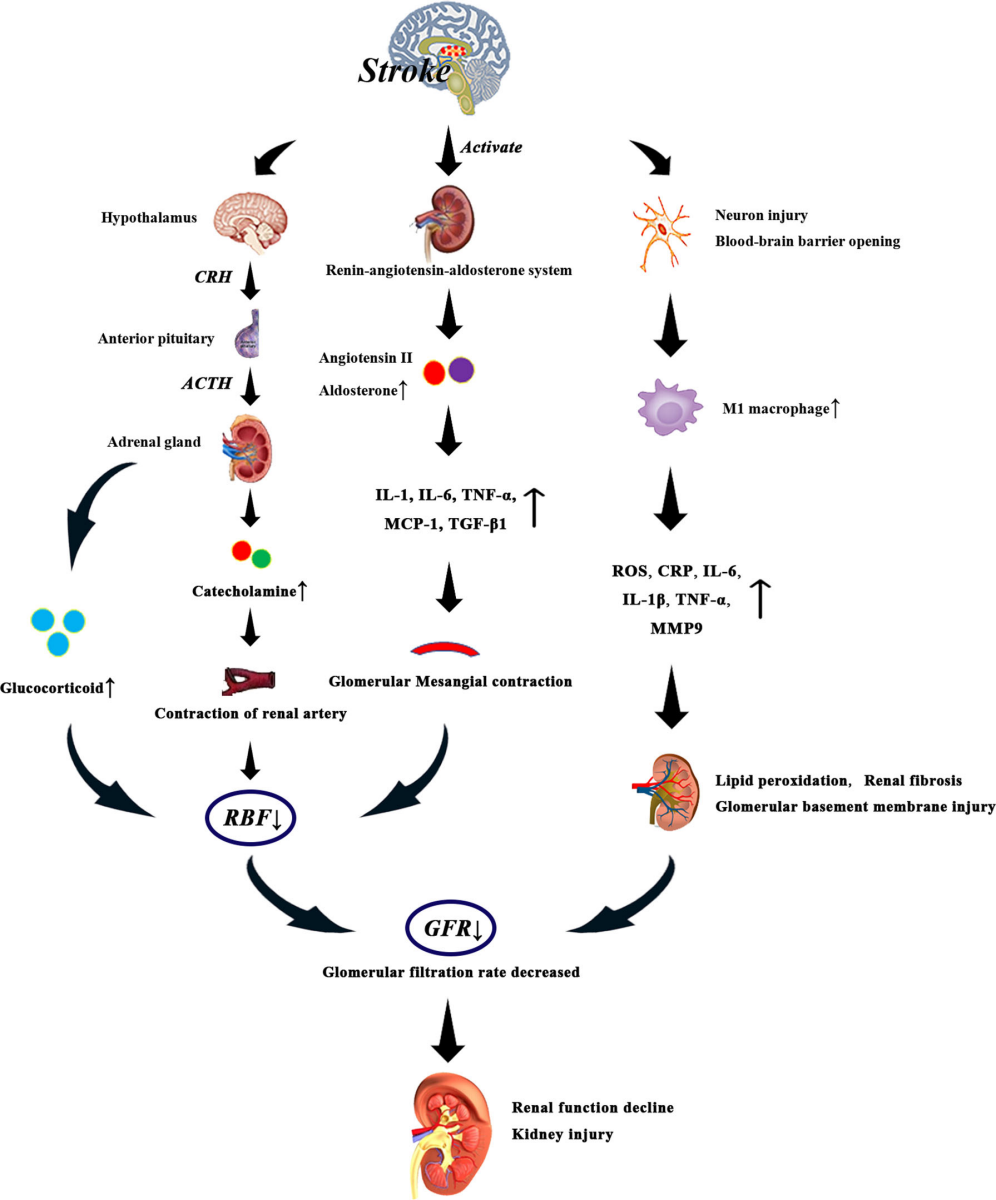
After stroke, the levels of glucocorticoid, catecholamine, angiotensin II and aldosterone increased due to the activation of HPA axis and renin-angiotensin-aldosterone system. Especially when the level of glucocorticoid is too high, the renal blood flow decreases significantly, which leads to the decrease of glomerular filtration rate. Catecholamine and angiotensin II bind to renal artery receptors and promote renal artery contraction and renal ischemia. Activation of renin-angiotensin-aldosterone system can promote systemic and glomerular capillary hypertension, and the direct fibrogenic and pro-inflammatory effects of angiotensin II and aldosterone may also lead to renal injury. In addition, angiotensin II can increase the levels of IL-1, IL-6, tumor necrosis factor-α and monocyte chemoattractant protein-1, thus reducing glomerular blood flow. On the other hand, after stroke, neurons are damaged, the blood-brain barrier is destroyed, and the production of M1 macrophages is increased, which induces the production of inflammatory factors such as C-reactive protein, IL-6, IL-1 β, tumor necrosis factor and matrix metalloproteinase-9, which leads to renal injury. These events eventually lead to decreased glomerular filtration rate, decreased renal function, and irreversible kidney damage.

After stroke, the hypothalamus-pituitary-adrenal axis can be activated to regulate the release of glucocorticoids from the adrenal gland. High levels of glucocorticoids can directly affect the function of glomeruli and renal tubules, resulting in the decrease of GFR (112). At the same time, elevated glucocorticoid levels can lead to vascular and hemodynamic changes, which indirectly affect renal blood flow (RBF) and glomerular function (112) (**Figure 3**). On the one hand, patients with acute brain injury due to excessive excitation of the sympathetic nerve, resulting in glomerular filtration function decreased, renal blood flow decreased (113). On the other hand, patients with acute cerebral ischemia often present with elevated catecholamines (114), and persistent excitation of the sympathetic nervous system leads to binding of catecholamines and angiotensin II to renal artery receptors, prompting renal artery contraction and renal ischemia (115). It can be seen that the increased levels of glucocorticoid and catecholamine may be related to the damage of renal function after stroke.

Activation of the renin-angiotensin-aldosterone system can promote systemic and glomerular capillary hypertension, and the direct fibrogenic and pro-inflammatory effects of angiotensin II and aldosterone may also lead to renal damage (116). Renin-angiotensin system (RAS) is involved in the pathogenesis of ischemic brain injury, and angiotensin II levels are increased in stroke patients (117). Angiotensin II can directly activate the expression of glomerular cytokines, inflammation and fibrosis factors, which has an effect on renal hemodynamics (118). In addition, angiotensin II also stimulates macrophage aggregation in glomeruli and tubular cells, increases the production of cytokines such as IL-1, tumor necrosis factor-α and monocyte chemoattractant protein-1, and acts on glomerular cells to promote the occurrence and development of glomerular injury. Angiotensin II induces the expression of TGF- β 1 and angiotensinogen genes, and induces the proliferation of renal interstitial fibroblasts through AT1 receptors, which may be involved in the pathogenesis of renal fibrosis (119). Another study showed that angiotensin II can induce the production of IL-6 in the kidneys. IL-6 can induce fibrosis gene expression and ET-1 gene expression, which leads to renal injury (120). Excessive activation of the sympathetic nervous system after stroke increases the release of Antidiuretic hormone (ADH), resulting in glomerular Mesangial contraction, glomerular blood flow reduction (121), resulting in renal function damage.

##### 1.3.1.2 Inflammatory and Immune Response

Immune and inflammatory responses play an important role in the progression of stroke and are also important causes of AKI and CKD (84). Macrophages are significant mediators of inflammation and immune regulation, and classical pro-inflammatory M1 macrophages are associated with renal disease (118). Macrophage-derived cytokines (ROS, IL) and inflammatory factors such as C-reactive protein (CRP), IL-6, IL-1 β, TNF- α and matrix metalloproteinase-9 (MMP9) after brain injury are related to renal injury (118). CRP is an acute inflammatory protein, mainly synthesized by hepatocytes, which increases 1000-fold in the site of infection or inflammation (122). CRP levels increased after ischemic stroke (123), and high levels of CRP decreased glomerular filtration rate (124). Reactive oxygen species (ROS) levels increase rapidly in the acute phase of stroke and enter the bloodstream through the blood-brain barrier (BBB) (125). Reactive ROS can cause different types of cell damage, especially lipid peroxidation and membrane damage (126). In the kidney, ROS mainly degrades the glomerular basement membrane and changes the function of glomerular and tubular cells (126). Microglia and macrophages produce and secrete IL-1 β after stroke (127). Il-1β can lead to renal injury and renal fibrosis (128). The expression of MMP-9 is increased during cerebral ischemia, which leads to neuronal injury, apoptosis and opening of BBB (129). It plays a special physiological role in the main cells of renal collecting duct (130). Another experimental study in rats has shown that increased circulating MMP-9 activity is associated with refractory albuminuria, which may lead to the progression of CKD in patients (131).

##### 1.3.1.3 Extracellular Vesicles and microRNA

In the kidney, EVS can come from blood cells, endothelial cells, podocytes or renal tubular epithelial cells (132). EVS may be a biomarker of kidney disease, which is related to inflammation, thrombosis and immunosuppression (132). The production of EVS after brain injury affects the normal physiological function of the kidney. EVS is related to the pathogenesis of acute renal injury and chronic renal disease, including AKI, CKD, renal fibrosis and various glomerular diseases (37). No matter what kind of dialysis treatment is performed, the level of platelet-derived EVS is increased, indicating that dialysis does not clear EVS (38). However, as far as the current research is concerned, the mechanism of EVS causing renal function damage through brain-kidney interaction is not clear. MiRNA is a small non-coding RNA molecule that regulates gene expression and participates in the occurrence and development of tubulointerstitial sclerosis and glomerular lesions (133). The level of miR expression changes after stroke. An experimental study in a mouse model of unilateral ureteral obstruction showed that TGF- β induced up- regulation of miR-21 expression, which was mediated by Smad2 signal, thus promoting renal fibrosis (134). Another *in vitro* study reported that astragaloside IV (AS-IV) improved renal function and renal fibrosis by inhibiting podocyte dedifferentiation and Mesangial activation induced by overexpression of miR-21 (135). It can be seen that the inhibition of Smad2/miR-21 signal pathway may be used as a new treatment to inhibit renal fibrosis in the future. In addition, miR-29c is the characteristic miRNA in diabetes. By knocking down miR-29c with specific antisense oligonucleotides, the proteinuria and glomerular Mesangial matrix in diabetic mice are significantly decreased (136).

#### 1.3.2 Treatment

When we treat stroke patients with renal injury, we need to evaluate the effectiveness and risk of intravenous thrombolysis and anticoagulation therapy. Studies have shown that stroke patients receiving intravenous injection of rt-PA increase the risk of decreased glomerular filtration rate and cerebral hemorrhage (137). Because the new oral anticoagulants apisaban and rivasaban are excreted mainly or partly by the kidneys, the half-life is prolonged in CKD patients, which not only enhances the antithrombotic effect, but also increases the risk of bleeding (138). There are no significant benefits of aspirin as a traditional antiplatelet drug in the treatment of stroke patients, but studies have shown that aspirin treatment of CKD patients has a greater absolute reduction in major cardiovascular events and mortality, this benefit seems to outweigh the increased risk of massive hemorrhage (139). Many anticoagulants and thrombolytic agents are used clinically to treat stroke, which increases the risk of use when patients are complicated with renal insufficiency. One of the reasons is that we do not know enough about the mechanism of brain-kidney interaction. Further exploration of this interaction will help us to find drugs that have dual protective effects on the brain and kidney.

### 1.4 Spleen Injury After Stroke

In the human body, the spleen, as one of the most important immune organs, has innate and acquired immune function and plays a vital role after stroke. After stroke, the brain broke out a serious inflammatory cascade reaction. Due to the role of chemokines and cytokines, the brain recruited a large number of spleen-derived immune cells to the brain injury site to combat the inflammatory response (4, 140). Many studies have found that shortly after stroke, the spleen shrinks sharply and the number of cells in the spleen decreases accordingly (74, 140–142). This change may reflect the increase of immune cell outflow from spleen to peripheral circulation and the increase of spleen cell group death (143). In one study, carboxyfluorescein diacetate succinimidyl ester (CFSE) was used to label the migration of splenocytes after cerebral ischemia. It was proved that after cerebral ischemic injury, NK cells, T cells, monocytes and NK cells entered the systemic circulation and migrated to the brain, aggravating brain injury (144). In addition, due to the role of cytokines and chemokines, IL-6, IFN- γ, TNF-α, MCP-1 and other molecules are recruited to the brain injury site and aggravate the brain injury (145, 146). At present, the exact mechanism of spleen activation after stroke is not clear, but the activation of sympathetic nervous system, antigen presentation of central nervous system and the production of chemokines have been proved to be important factors (143).

#### 1.4.1 Pathophysiological Mechanism of Spleen Injury After Stroke

##### 1.4.1.1 Sympathetic Nervous System

Shortly after stroke, activation of the sympathetic nervous system leads to an increase in norepinephrine and epinephrine in the systemic circulation. In rodents, the spleen shrinks after MCAO, which may be due to the expression of α-1 adrenergic receptor in the splenic sac of rats, which leads to splenic contraction after activation (141). Both norepinephrine and epinephrine have been shown to cause significant splenic atrophy (147). Prazosin or carvedilol could prevent the spleen from shrinking, but only carvedilol could significantly reduce the infarct volume (148). These results suggest that α and β adrenergic receptors seem to mediate the response of the spleen to stroke.

##### 1.4.1.2 Antigen Presentation of the Central Nervous System

In the early stage after stroke, the activation of danger-associated molecular patterns (DAMP) causes the damaged brain to secrete various antigens such as ATP, high mobility group protein 1 (HMGB1), heat shock protein (HSP) and nicotinamide adenine dinucleotide (NAD) (149). These antigens interact with antigen-presenting cell receptors to activate innate and acquired immune responses, eventually recruiting immune cells to the injured brain (150).

##### 1.4.1.3 Production of Cytokines and Chemokines

After MCAO, the levels of TNF-α, IFN-γ, IL-6, MCP-1 and IL-2 secreted by mouse splenocytes increased significantly (151). In addition, IFN γ can also activate the expression of chemokine interferon-inducible protein 10, leading to neurodegeneration (40). In cerebral ischemic injury, CCL2 (MCP-1) mediates monocyte and neutrophil infiltration. Inhibition of CCL2/CCR2 axis can reduce brain edema and leukocyte infiltration to improve the results of cerebral reperfusion (41). The treatment of MCAO mice with the antibacterial drug moxifloxacin (MFX) significantly reduced the expression of CCR2 in spleen tissue and brain after ischemia, and reduced the area of cerebral infarction (152). Other cytokines, such as CCL3, CCL5 and CXCR4-CXCL12, have been shown to play a role in splenic response after stroke (153).

#### 1.4.2 Treatment

Some experiments have proved that compared with the rats without splenectomy two weeks before permanent middle cerebral artery occlusion, the area of cerebral infarction was significantly reduced and the number of macrophages, activated microglia and neutrophils was also greatly reduced (154). However, it is obvious that splenectomy can’t be used as a practical clinical stroke treatment, because from a long-term point of view, the risk of infection caused by failure to maintain normal immune function after splenectomy is greatly increased. Therefore, drugs and cell-based therapy for the interaction between the peripheral immune system and the brain may be used as stroke treatment options (155, 156). There is growing evidence that intravenous injection of various types of stem cells can reduce neurological damage caused by stroke to a greater extent than intracerebral administration (157). Intravenous infusion of human umbilical cord blood cells (HUCB) to stroke patients can improve the cerebral ischemic microenvironment and restore neurological function (142, 158). Transplantation of HUCB cells after MCAO increased the production of anti-inflammatory cytokines IL-10, decreased the production of inflammatory cytokines TNF- α and interferon-γ, and inhibited the proliferation of splenic CD8+T cells (157). In addition, multipotential adult progenitor cells (MAPC) treatment can increase the number of Treg cells in the spleen, up-regulate the level of serum IL-10, reduce the release of IL-1 β and IL-6 from splenocytes, and restore the reduction of spleen mass caused by stroke (159). These evidences suggest that spleen is a key target for MAPC to regulate immune response, regulate local cerebral microenvironment and promote rehabilitation after stroke (159).

### 1.5 Gastrointestinal Bleeding After Stroke

Stroke cuts off the connection between the central nervous system and the gastrointestinal system, resulting in dysphagia, gastrointestinal bleeding, delayed gastrointestinal emptying, etc (160). Dysphagia and gastrointestinal bleeding (GIB) are common complications after stroke and are related to poor prognosis. Since dysphagia has been described in post-stroke pneumonia, we will not repeat it here.

GIB is a common complication in patients with acute stroke and may affect stroke treatment, such as antiplatelet or anticoagulation therapy (161). The incidence of gastrointestinal bleeding after stroke is 1.5%-7.8%, which may be related to the subtype of stroke (162, 163). Patients with gastrointestinal bleeding after stroke were characterized by sudden hematemesis, decreased hemoglobin or orthostatic hypotension. Advanced age, disturbance of consciousness, severe neurological impairment, infection and posterior circulation infarction are independent risk factors for GIB in patients with acute stroke, and GIB is also a high risk factor for death within 1 year after acute stroke (164). At present, clinical trials have demonstrated that AIS-GIB score is an effective clinical grading standard for predicting GIB during hospitalization after acute ischemic stroke, which plays a certain role in helping the incidence of GIB and improving prognosis after AIS (42).

#### 1.5.1 Pathophysiological Mechanism of GIB

At present, the pathogenesis of GIB after stroke is not fully understood. Stress ulcer caused by acute brain injury, increased gastric acid secretion or mucosal ischemia caused by overactivity of vagus nerve may be one of the causes of GIB.

Aspirin is a commonly used antiplatelet drug in patients with stroke, and its low-dose side effects may also cause gastrointestinal mucosal damage, leading to gastroduodenal ulcer (165). A meta-analysis shows that cilostazol is less likely to have gastrointestinal bleeding than aspirin, although other gastrointestinal adverse reactions are more likely to occur (166). In addition to stress ulcer and antiplatelet effect, systemic inflammation and oxidative stress may be the pathophysiological mechanism of gastrointestinal mucosal injury after stroke (167). During ischemic stroke, ulcers can also be formed due to the decrease of gastric mucosal blood flow. Some animal experimental studies have shown that norepinephrine neurons reduce gastric mucosal blood flow through α-adrenergic receptors in rats with cerebral ischemia, resulting in damage to the integrity of gastric mucosa. Vagotomy can eliminate the decrease of gastric mucosal blood flow during cerebral ischemia (168). In addition, malnourished stroke patients are also more likely to develop gastrointestinal bleeding (169).

#### 1.5.2 Treatment

The treatment and prevention of stroke patients with gastrointestinal bleeding should follow routine guidelines. ASA (antiplatelet-statin-antihypertensive) is a drug used for secondary stroke prevention to prevent cerebrovascular thrombosis in patients with cerebrovascular disease or patients at risk of cerebrovascular disease (160). However, antiplatelet drugs should be carefully selected when treating this type of patients, and their anti-platelet and gastrointestinal mucosal damage may increase the risk of bleeding. Therefore, antiplatelet therapy for secondary stroke prevention must be individualized according to the patient’s complications, including the risk of bleeding (170). Studies have shown that antiplatelet drugs in combination with proton pump inhibitors (PPI) or misoprostol can reduce the risk of gastrointestinal injury (171). PPI can effectively inhibit the secretion of basal gastric acid and stimulating gastric acid, and make the PH of gastric juice more than 6.0. therefore, it can induce hemostasis by body fluid and platelet. In addition, PPI can also promote gastric mucosal blood, improve local microcirculation, accelerate mucosal regeneration and repair, and further control bleeding.

## 2 Conclusion

Peripheral organ injury and dysfunction are very common after stroke, which usually occur within one week after stroke, so measures need to be taken to prevent and treat them in time. The most common complications after stroke include pulmonary infection, heart failure, acute renal injury and gastrointestinal bleeding. Understanding the pathogenesis, high risk factors and onset time of these complications is helpful for early diagnosis and treatment. The interaction between brain and peripheral organs after stroke is almost always carried out through humoral regulation and neuroregulation. The activation of the immune system and inflammatory response are very important for stroke. They affect many peripheral organs and thus affect the outcome of stroke. Here, we also emphasize and discuss the role of immune and inflammatory responses in all the organs under discussion, hoping to clarify the relationship between them and stroke. However, there are still many problems that have not yet been overcome. The injury of peripheral organs after stroke is usually not caused by the interaction of two or several systems, but by the participation of multiple systems throughout the body. There is no experimental model that can summarize all aspects at present. Elucidating the interaction between central and peripheral organs and peripheral organs will help us to develop more effective treatments.

## Author Contributions

JW and JZ wrote the initial draft. YY contributed to reviewing the literature. QX and YL prepared the figures and table. SF and XX collected the literature. LG and ZJ designed the manuscript and prepared the final version. The authors read and approved the final manuscript.

## Funding

This study was supported by the National Natural Science Foundation of China (Nos. 82071339 and 81771283) and the Natural Science Foundation of Hubei Province (Nos.2020CFB613).

## Conflict of Interest

The authors declare that the research was conducted in the absence of any commercial or financial relationships that could be construed as a potential conflict of interest.

## Publisher’s Note

All claims expressed in this article are solely those of the authors and do not necessarily represent those of their affiliated organizations, or those of the publisher, the editors and the reviewers. Any product that may be evaluated in this article, or claim that may be made by its manufacturer, is not guaranteed or endorsed by the publisher.

## Glossary

**Table:**

ACEI	Angiotensin converting enzyme inhibitors
ADH	Antidiuretic hormone
AIS	Acute ischemic stroke
AKI	Acute kidney injury
ALIAS	Albumin in Acute Ischemic Stroke
ARDS	Acute respiratory distress syndrome
AREST	Apixaban for Early Prevention of Recurrent Embolic Stroke and Hemorrhagic Transformation
ASA	Antiplatelet-statin-antihypertensive
BBB	Blood-brain barrier
CAMP	Cyclic adenosine monophosphate
CAN	Autonomic neural network
CKD	Chronic kidney disease
COSSACS	Continue Or Stop post-Stroke Antihypertensives Collaborative Study
CNS	Central nervous system
CRP	C-reactive protein
DAMPs	Damage-associated molecular patterns
EVS	Extracellular vesicles
GFR	Glomerular filtration rate
GIB	Gastrointestinal bleeding
HMGB1	High mobility group protein 1
HPA	Hypothalamus-pituitary-adrenal axis
HSP	Heat shock protein
HUCB	Human umbilical cord blood cells
ICH	Intracerebral hemorrhage
IL	Interleukin
IFN	Interferon
IRIS	Insulin Resistance Intervention after Stroke
MAPC	Multipotential adult progenitor cells
MCAO	Middle cerebral artery occlusion
MCP	Membrane cofactor protein
MFX	Moxifloxacin
MMP9	Matrix metalloproteinase-9
NAD	Nicotinamide adenine dinucleotide
NAVIGATE-ESUS	l New Approach Rivaroxaban Inhibition of Factor Xa in a Global trial versus acetylsalicylic acid to prevent embolism in Embolic Stroke of Undetermined Source
NIHSS	National Institutes of Health Stroke Scale
NMDA	N-methyl-D-aspartate
NPE	Neurogenic pulmonary edema
PCP	Pulmonary capillaryhydrostatic pressure
PPI	Proton pump inhibitors
RAS	Renin-angiotensin system
RBF	Renal blood flow
RT-PA	Recombinant tissue plasminogen activator
ROS	Reactive oxygen species
SAH	Subarachnoid hemorrhage
SAP	Stroke-associated pneumonia
SIIS	Stroke-induced immunosuppression
SVR	Systemic vascular resistance
TGF	Transforming growth factor
TGI	Transient global cerebral ischemia
TLR	Toll-like receptor
TMAO	Trimethylamine N-oxide
TNF	Tumor necrosis factor
URICO-ICTUS	Efficacy Study of Combined Treatment With Uric Acid and r-tPA in Acute Ischemic Stroke
